# eHealth Literacy in a Sample of South Asian Adults in Edmonton, Alberta, Canada: Subanalysis of a 2014 Community-Based Survey

**DOI:** 10.2196/29955

**Published:** 2022-03-30

**Authors:** Mark J Makowsky, Shahnaz Davachi, Charlotte A Jones

**Affiliations:** 1 Faculty of Pharmacy and Pharmaceutical Sciences, University of Alberta Edmonton, AB Canada; 2 Primary Health Care, Alberta Health Services Calgary, AB Canada; 3 Faculty of Medicine, Southern Medical Program, University of British Columbia Okanagan Campus Kelowna, BC Canada

**Keywords:** eHealth literacy, consumer health information, ethnicity, cross-sectional survey, Canada, digital health, eHealth, ePatient, health technology, cardiovascular disease, diabetes, sociodemographics, mobile phone

## Abstract

**Background:**

Digital health interventions are efficient and flexible methods for enhancing the prevention and management of cardiovascular disease and type 2 diabetes. However, little is known about the characteristics associated with eHealth literacy in the Canadian South Asian population.

**Objective:**

The aim of this study is to describe perceived eHealth literacy and explore the extent to which it is associated with sociodemographic, health status, and technology use variables in a subset of South Asian Canadians.

**Methods:**

We analyzed data from the e-Patient Project survey, a mixed-mode cross-sectional survey that occurred in 2014. The eHealth Literacy Scale (eHEALS) was used to measure eHealth literacy in a convenience sample of 511 English- or Punjabi-speaking South Asian adults recruited from a community pharmacy, a family physician office, and community events in Edmonton, Alberta. Multivariable quantile regression was used to explore variables associated with eHealth literacy.

**Results:**

The analysis was restricted to 301 internet users (mean age 39.9, SD 14.8 years; 166/301, 55.1% female) who provided responses to all 8 eHEALS questions and complete demographic information. The mean overall eHEALS score was 29.3 (SD 6.8) out of 40, and 71.4% (215/301) agreed to at least 5 out of the 8 eHEALS items. The eHEALS item with the lowest level of agreement was “I can tell high-quality health resources from low-quality health resources on the internet” (182/301, 60.5%). Although there were statistically significant differences in eHEALS scores according to age, educational achievement, language preference, and the presence of chronic medical conditions, multivariable regression analysis indicated that language preference was the only variable independently associated with eHealth literacy (coefficient –6.0, 95% CI –9.61 to –2.39).

**Conclusions:**

In our sample of South Asian Canadian internet users, preference for written health information in languages other than English was associated with lower eHealth literacy. Opportunities exist to improve eHealth literacy using culturally and linguistically tailored interventions.

## Introduction

### Background

Digitally engaged patients are those who have taken up new digital media technologies in their own medical care and preventative health efforts [1]. These eHealth technologies typically support self-care and include internet-based resources where lay people can seek information about health, web-based communities supported by social networking sites, mobile health (mHealth)—the practice of medicine and public health with support from mobile devices, telemedicine and remote patient monitoring where patients communicate with health care providers via technology instead of face-to-face communication, and others [1-3]. Emerging evidence suggests that eHealth-and mHealth-based interventions can improve the prevention and management of chronic health conditions [4,5]. For example, systematic reviews suggest the potential for the improvement of cardiovascular lifestyle-related risk factors [6] including weight loss [7-9], physical activity [10,11], and management of diabetes [12-14].

To optimally engage with and have equitable access to a digital health care environment, health care consumers must have an understanding of their condition as well as the skills to effectively use electronic resources [15-17]. This is referred to as eHealth literacy, which is defined as the ability to seek, find, understand, and appraise health information from electronic sources and apply the knowledge gained to address or solve a health problem [18]. There are six main domains of eHealth literacy: health, computers, media, science, and information literacy, as well as traditional literacy and numeracy [18]. eHealth literacy has taken on greater importance as the COVID-19 pandemic has shifted primary care from office visits to telephone or video care in the Canadian health care system [19] and resulted in a broader *infodemic* of medical misinformation [20,21]. Assessing eHealth literacy is important because it has been associated with positive outcomes from internet searches in health knowledge and information gathering, self-management of health needs and health behaviors, and interactions with physicians [22-25]. eHealth literacy is modifiable, and several studies have shown that it can be improved in older individuals and those with chronic health conditions [15,26,27].

South Asian Canadians are a large visible minority group [28] who face a high prevalence of cardiovascular disease and diabetes, both of which are conditions for which self-management are required [29-31]. Expanded access to and use of culturally appropriate eHealth strategies may assist South Asian Canadians in addressing documented gaps in risk factors and diabetes control [30] by addressing documented barriers [32,33] such as language, sociocultural factors, misconceptions around diet and physical activity, lack of access to culturally tailored diet counseling, and compliance with pharmacotherapy.

Whether South Asian Canadians have adequate eHealth literacy to effectively use eHealth to improve their health is uncertain as are the demographic and technology use determinants of eHealth literacy in this community. Although eHealth strategies may support improving both the prevention and management of cardiovascular disease and type 2 diabetes, a systematic review of eHealth literacy suggests that underserved populations may have lower levels of competence in the 6 core domains of eHealth literacy, decreased access to health infrastructure, and technology [34]. For example, research conducted by Statistics Canada suggests that immigrants to Canada originating from non–English- and non–French-speaking countries score below the national average in health literacy. On the basis of 2003 survey data, fewer immigrants (24%) than nonimmigrants (44%) had requisite levels of health literacy [35]. Research on Danish immigrants has suggested that descendants and immigrant women have lower levels of eHealth literacy and health literacy than women of Danish origin [36]. In contrast, data on immigrants to Israel suggest that language barriers because of immigration, negatively impact health literacy but have no impact on eHealth literacy [37]. Research on primary digital divides in internet access by race or ethnicity has been conflicting, with some suggesting no difference in digital use divides [38], whereas others suggest that they do exist [39]. Demographic and socioeconomic variables associated with eHealth literacy in the literature are inconsistent and are suggested to be population-dependent [17].

### Objectives

Owing to a lack of information on digital device use and eHealth literacy in South Asian Canadians, we conducted the South Asian e-Patient Project, a survey conducted in a convenience sample of 831 South Asian Canadians in Edmonton, Alberta in 2014 [40]. We previously reported that engaging members of this community via eHealth interventions is feasible. However, we found evidence of digital divides in the use of the internet, digital devices, and apps for health purposes by language preference, education, age, gender, confidence in filling out medical forms, and the number of years lived in Canada. Further description and examination of the characteristics associated with eHealth literacy in members of the Canadian South Asian Community is important because this information could be used to identify levels of readiness for eHealth, to inform and justify the development of tailored solutions to overcome identified gaps in the required skills to effectively apply web-based health information and other mHealth interventions and to inform and assist health care providers to optimally engage individuals in remote internet-based care and with existing web-based health information resources. Therefore, the objectives of this study are to (1) describe levels of eHealth literacy among a subset of South Asian adult internet users who were invited to complete the eHealth Literacy Scale (eHEALS) as part of the community-based e-Patient Project survey and (2) explore sociodemographic, health status, and technology use variables and their association with eHealth literacy among survey respondents.

## Methods

### e-Patient Project Study Design

The design as well as the results on the prevalence and variables associated with internet and digital device use for health purposes from the e-Patient Project survey have been published previously [40]. Briefly, the survey was an anonymous, mixed-mode survey conducted with a convenience sample of English- or Punjabi-speaking South Asian adults between May 18 and August 31, 2014. We used a community-based approach and worked in partnership with 13 faith-based, cultural, community, and health care organizations in Edmonton, Alberta. The survey was designed to evaluate levels of engagement with the internet, digital device ownership, use of health and fitness apps, health information–seeking practices, preferences for delivery modalities for future eHealth interventions, and eHealth literacy. (Multimedia Appendix 1) The survey was primarily a computer-assisted in-person interview conducted at faith-based gathering places, health care settings, community centers, and events using the Qualtrics (Qualtrics Corporation) web-based survey platform. Most questions were adopted from existing survey instruments, including the Pew Research Center’s Internet & American Life Project 2012 Health survey [41-43] and the 2012 Statistics Canada Canadian Internet Use survey [44]. The survey was pilot-tested with 19 individuals from the target communities.

### eHealth Literacy Assessment—Inclusion Criteria, Recruitment, and Data Collection

A subset of the survey participants was invited to complete the eHealth literacy assessment. Owing to the time required to complete the survey, this subset included all individuals recruited in person at the participating community pharmacy, family physician practice, a large community event, and 1 community center, as well as those who completed the survey via the web. Individuals recruited at faith-based gatherings were excluded from the eHealth literacy assessment.

Potential respondents were notified about the study via personal invitations by pharmacy or clinic staff, announcements at community events, and posters. We recruited consecutive attendees from the community pharmacy and family physician office sites. Individuals were then approached by trained, bilingual English- and Punjabi-speaking research assistants or community volunteers. They were presented with the e-Patient Project survey information letter and were asked if they would like to participate. The agreement and completion of the survey implied participants’ consent.

Research staff administered the survey, including the eHealth literacy assessment, via computer-assisted personal interviews in English or Punjabi, according to respondent preferences. One-on-one interviews using paper surveys were conducted at the community event before the tablet computers became available. A web-based version was also offered to those who preferred to complete the survey on their own time. Respondents who completed the survey were offered a reusable shopping bag and the opportunity to enter a draw for a tablet computer or various gift cards as incentives.

### Measurement of eHealth Literacy

eHealth literacy was measured using the 8-item eHEALS scale, which is the most commonly used validated measure of eHealth literacy [45,46]. The eHEALS measures the concept of eHealth literacy, defined as a set of skills required to effectively engage information technology for health and has shown high levels of internal consistency and test-retest reliability [46]. Each of the 8 items is rated on a 5-point Likert scale (1=strongly disagree to 5=strongly agree). The overall score ranges from 8 to 40, with higher scores suggesting higher eHealth literacy. In addition, 2 supplemental eHEALS items, which do not contribute to the overall score, were also included before the 8 items to measure the perceived usefulness of the internet to help make health decisions and the perceived importance of being able to access health resources on the internet.

We created a Punjabi version of the survey and the eHEALS instrument according to the World Health Organization guidelines for translation and adaptation of instruments [47] (Multimedia Appendix 2). One Punjabi-speaking translator with a medical background who was also fluent in English conducted a forward translation from English to Punjabi. Emphasis was placed on conceptual rather than literal translation. A panel of 2 bilingual community member reviewers further reviewed and identified inadequate expressions and concepts in the translated versions. The back translation was conducted by a separate translator who was fluent in both English and Punjabi. The translated eHEALS was included in the pilot test, and translation discrepancies were discussed and addressed by the project team. The internal consistency for the 8 item eHEALS for the 301 respondents was Cronbach *α*=.950, and principal components analysis produced a single factor solution with factor loadings from 0.68 to 0.80 among the 8 items, with eigenvalue=5.95 and 74.4% of the variance explained (Multimedia Appendix 3).

### Measurement of Sociodemographic and Internet Use Variables

Demographic factors included age, sex, education, marital status, duration of time lived in Canada, and South Asian community affiliation. Individuals who answered affirmatively to either *Do you go online at least occasionally* or *Do you send or receive email at least occasionally*? were characterized as internet users. Language preference was determined by asking *In what language would you prefer to receive written health information* and the categories were collapsed into includes English or does not include English. We estimated health literacy using the question *How confident are you filling out medical forms by yourself?* that was effective in detecting inadequate health literacy as measured using the Short Test of Functional Health Literacy in Adults [48]. Individuals who indicated being likely or very likely to use at least one of six different modes of eHealth interventions in the next 12 months were deemed to be interested in eHealth interventions.

### Statistical Analysis

We limited the analysis to internet user respondents who provided complete responses to all 8 eHEALS questions and complete demographic information for the planned multivariate analysis. Surveys with other missing data were included in the analysis, and descriptive statistics for categorical variables were depicted as proportions of total cases, including those with missing data. SPSS (version 28; IBM Corp) was used to compute descriptive statistics for the demographic characteristics, technology use variables, and eHEALS scores. Summary eHEALS scores were calculated by summing the responses to 8 eHEALS questions. We also explored the proportion of individuals who agreed to at least five out of the eight eHEALS items, an indicator of adequate eHealth literacy [49], and the number of participants scoring 26 or more, which has been considered an indicator of high eHealth literacy [17,50]. We explored bivariate differences in eHEALS scores across demographic, health status, and technology use outcome variables using the 2-tailed Welch *t* test or Welch *F* test as appropriate [51-57]. Statistical significance was established at *P*<.05 and Bonferroni correction was used for post hoc tests.

Multivariable quantile regression was performed using R (version 3.1.3; The R Foundation) to assess the effect of each demographic variable on the eHEALS score while controlling for the effect of others. This analysis was restricted to internet users and explored both demographic and internet use predictors. Quantile regression through the median was performed instead of mean regression [58] because regular regression residuals differed substantially from normality and violated the constant error variance assumption as well. Log and square root transformations also did not work. Quantile regression models conditional quantiles instead of conditional means. Here, we model the conditional median (the 0.5th quantile). It does not assume normality and constant error variance and is robust against outliers. The CIs and *P* values reported were based on bootstrap SEs with 1000 repetitions.

### Ethics Approval

The Health Research Ethics Board at the University of Alberta (Pro00038210) approved this study.

## Results

### Participant Flow

**Figure 1:**
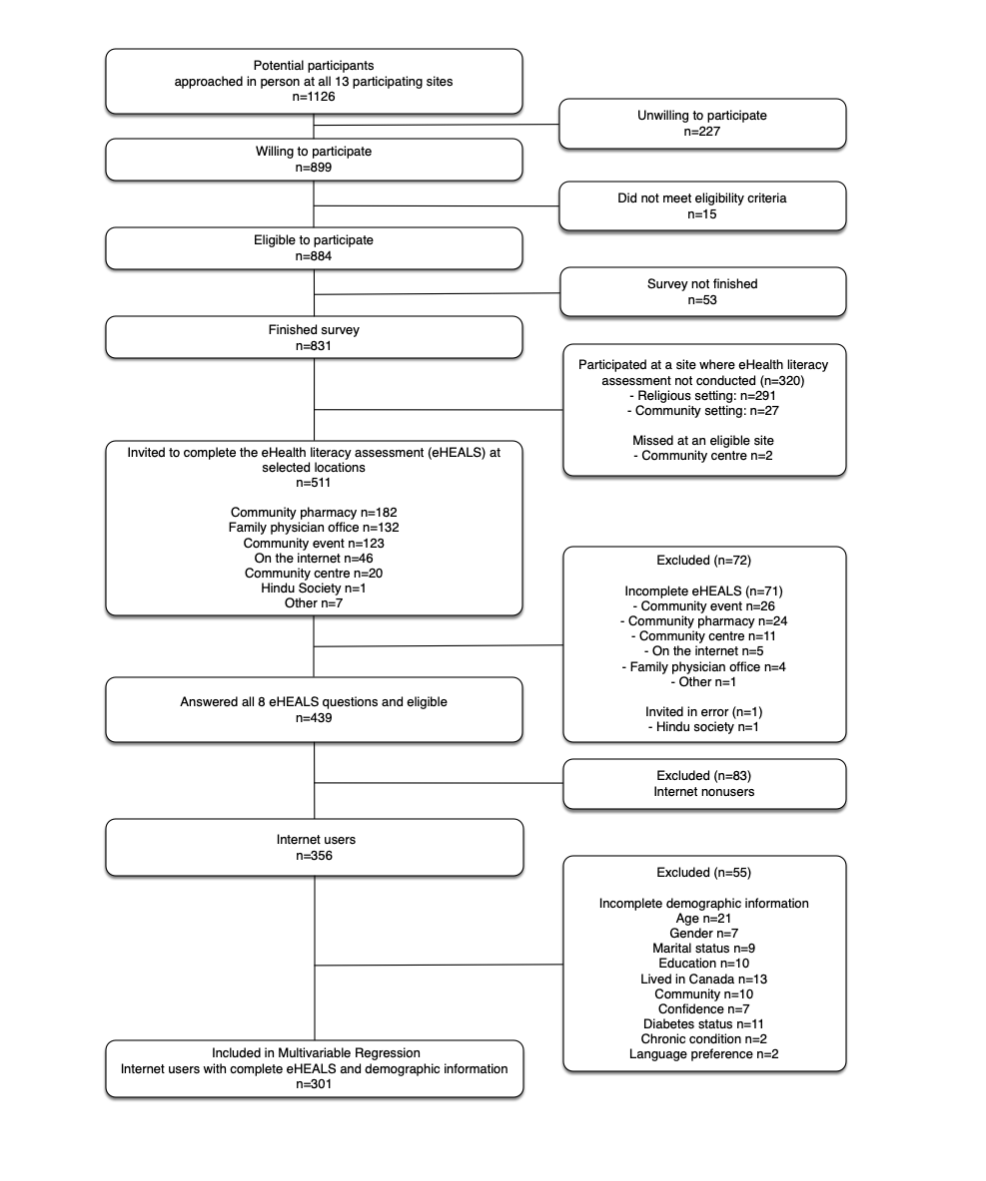
Flow diagram of the eHealth Literacy Scale assessment in the e-Patient Project Survey, Edmonton, Alberta, 2014.

A total of 511 individuals were invited to complete the eHealth Literacy Assessment. Although we originally invited both internet users and noninternet users to complete the eHEALS assessment, given that it is more meaningful to investigate eHealth literacy in internet users, in this paper, we focus on reporting the results in the n=301 internet users who provided data for all 8 HEALS items and demographic characteristics. (Figure 1). Findings from the 83 noninternet user eHEALS responders are shown in Multimedia Appendix 4.

### Participant Characteristics

Table 1 shows the demographic characteristics and the mean eHEALS scores of the study sample**.** The mean age was 39.9 (SD 14.8) years and just over half of the respondents were female (166/301, 55.1%). Most respondents were married (235/301, 78.1%); had college, university, or higher education (238/301, 79.1%); and self-identified as Sikh (212/301, 70.4%). A total of 29.2% (88/301) lived in Canada for <5 years. Most respondents (260/301, 86.4%) indicated that English was their preferred language for written health information. Overall, 81.8% (246/301) indicated that their health was at least good, 53.2% (160/301) had at least 1 of the 8 chronic health conditions we inquired about, and only 1.7% (5/301) reported not being confident at all in filling out medical forms by themselves. Most respondents (194/301, 64.5%) were recruited from the participating community pharmacy and family physician office.

**Table 1:** Demographic characteristics of respondents and mean overall eHEALS^a^ scores among South Asian study participants by demographic characteristics.

Demographic		Values (n=301), n (%)	eHEALS score, mean (SD)	Difference in eHEALS scores (95% CI)	*P* value^b^	
**Age (years)**						*.*009^c^
	<65	275 (91.4)	29.68 (6.55)	Referent		
	≥65	26 (8.6)	24.92 (8.40)	–4.76 (–8.23 to –1.29)		
**Sex**						*.*14^c^
	Male	135 (44.9)	28.61 (7.79)	Referent		
	Female	166 (55.1)	29.81 (5.92)	1.21 (–2.81 to 0.40)		
**Marital status**						*.*12^c^
	Not married	66 (21.9)	30.55 (7.65)	Referent		
	Married	235 (78.1)	28.91 (6.57)	–1.63 (–3.69 to 0.42)		
**Education**						*.*01^d^
	<High school	3 (1)	15.67 (12.42)	Referent	N/A^e^	
	High School	60 (19.9)	25.27 (7.91)	9.60 (–31.32 to 50.52)	*.*51^f^	
	≥College	238 (79.1)	30.45 (5.88)	14.79 (–27.27 to 56.84)	*.*30^f^	
**Lived in Canada (years)**						*.*57^e^
	>5	213 (70.8)	29.41 (7.07)	Referent		
	0-5	88 (29.2)	28.94 (6.29)	–0.47 (–2.10 to 1.66)		
**South Asian community**						*.*16^d^
	Sikh	212 (70.4)	28.83 (7.28)	Referent	N/A	
	Hindu	42 (14)	30.40 (5.54)	1.57 (–0.79 to 3.94)	*.*26^f^	
	Other	47 (15.6)	30.26 (5.64)	1.43 (–0.87 to 3.72)	.31^f^	
**Confidence in filling out medical forms**						.35^g^
	>Not at all	296 (98.3)	29.36 (6.68)	Referent		
	Not at all	5 (1.7)	23.80 (13.55)	–5.57 (–19.06 to 7.83)		
**Health status**						.83^h^
	Excellent	43 (14.3)	28.23 (9.18)	Referent		
	Very good	70 (23.3)	29.81 (6.57)	1.58 (–2.92 to 6.08)		
	Good	133 (44.2)	29.50 (6.21)	1.27 (–2.96 to 5.50)		
	Fair	41 (13.6)	28.61 (6.95)	0.38 (–4.57 to 5.32)		
	Poor	14 (4.7)	29.50 (5.63)	1.27 (–4.62 to 7.16)		
**Diabetes**						.58^c^
	No	259 (86)	29.36 (6.94)	Referent		
	Yes	42 (14)	28.76 (6.25)	–0.59 (–2.71 to 1.52)		
**High blood pressure**						.02^c^
	No	241 (80.1)	29.9 (6.30)	Referent		
	Yes	55 (18.3)	26.95 (8.35)	–2.95 (–5.34 to –0.56)		
**Heart disease**						.51^c^
	No	279 (92.7)	29.5 (6.69)	Referent		
	Yes	12 (4)	27.5 (10.05)	–2.0 (–8.42 to 4.42)		
**Lung conditions**						.68^c^
	No	274 (91)	29.29 (6.87)	Referent		
	Yes	19 (6.3)	29.95 (6.50)	0.66 (–2.57 to 3.88)		
**Arthritis**						.14^c^
	No	261 (86.7)	29.49 (6.76)	Referent		
	Yes	31 (10.3)	27.29 (7.70)	–2.20 (–5.13 to 0.74)		
**Cancer**						.05^c^
	No	283 (94)	29.53 (6.73)	Referent		
	Yes	11 (3.7)	23.82 (8.54)	–5.71 (–11.48 to 0.06)		
**Other chronic condition**						.046^c^
	No	244 (81.1)	29.72 (6.83)	Referent		
	Yes	45 (15)	27.49 (6.75)	–2.23 (–4.42 to –0.04)		
**High cholesterol**						.16^c^
	No	193 (64.1)	30.15 (5.67)	Referent		
	Yes	41 (13.6)	28.24 (8.16)	–1.90 (–4.59 to 0.79)		
**≥1 Chronic condition^i^**						.08^c^
	No	160 (53.2)	29.93 (6.42)	Referent		
	Yes	141 (46.8)	28.52 (7.24)	–1.41 (–2.97 to 0.16)		
**Language preference**						<.001^f^
	English	260 (86.4)	30.38 (5.86)	Referent		
	Not English	41 (13.6)	22.22 (8.32)	–8.17 (–10.80 to –5.45)		
**Location of recruitment**						.03^h^
	Community setting	70 (23.3)	27.21 (8.21)	Referent	N/A	
	Health setting	194 (64.5)	30.07 (6.31)	2.86 (0.29 to 5.43)	.03^f^	
	Web-based	37 (12.3)	28.97 (5.93)	1.76 (–1.53 to 5.05)	.42^f^	

^a^eHEALS: eHealth Literacy Scale.

^b^*P* value is for the comparison of eHEALS scores.

^c^Welch *t* test.

^d^Welch *F* test on trimmed mean and Winsorized variance as data violated the constant variance assumption and were far from normal.

^e^N/A: not applicable.

^f^Pairwise comparison using the Games-Howell post hoc method; a *P* value of 0.5/number of comparisons was considered statistically significant.

^g^The bootstrap method with 1000 repetitions was used, as the data differed substantially from the normal distribution. For the bootstrap method, bias-corrected and accelerated CIs were reported.

^h^The Welch *F* test was used as the data violated the equal variance assumption of the analysis of variance.

^i^Chronic conditions included diabetes or sugar disease, high blood pressure, heart disease (eg, angina, heart attack, or stroke), lung conditions (eg, asthma or bronchitis), arthritis, cancer, high cholesterol, or other chronic conditions treated with daily medication.

### Overall eHEALS Scores and Patterns of Item Responses

**Figure 2:**
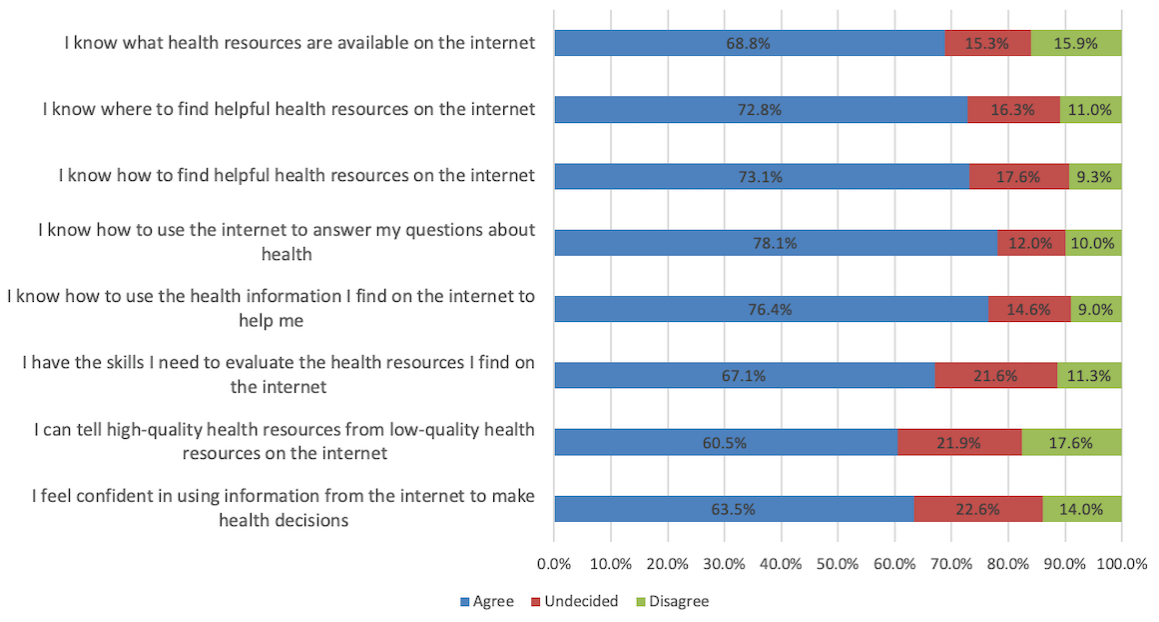
Frequency of responses to 8-item eHealth Literacy Scale among South Asian study participants (n=301 internet users). Data for strongly disagree and disagree were collapsed as was agree and strongly agree.

Total scores on the eHEALS ranged from 8 to 40 and were negatively skewed with a mean of 29.27 (SD 6.84), median of 31, and IQR of 27 to 32. A total of 2.3% (7/301) participants had the worst possible score, whereas 4.7% (14/301) of participants had the best possible score. Over three quarters of respondents (253/299, 84.6%) felt it is important to be able to access health resources on the internet and that the internet is useful in helping make decisions about their health (234/298, 78.5%). The proportion of respondents who agreed with each eHEALS item is shown in Figure 2. Almost three quarters of respondents (215/301, 71.4%) had adequate health literacy (ie, agreed to at least 5 out of the 8 eHEALS items) and 78.1% (235/301) had eHEALS scores ≥26. The 2 items with the lowest levels of agreement were for *I can tell high-quality health resources from low-quality health resources on the internet* (182/301, 60.5%) and *I feel confident in using information from the internet to make health decisions* (191/301, 63.5%).

### Mean eHEALS Scores by Demographic and Health Status Characteristics

Exploratory bivariate analysis showed that mean eHEALS scores were significantly lower in those aged ≥65 years (29.68, SD 6.55 vs 24.92, SD 8.40; difference –4.76, 95% CI –8.23 to –1.29; *P=*.01), and age was negatively correlated with eHEALS scores (Spearman correlation coefficient=–0.188; *P*=.001). Further, there were statistically significant higher mean eHEALS scores in those with college or university education versus high school education (30.45, SD 5.88 vs 25.27, SD 7.91; difference 5.19, 95% CI 2.58 to 7.79; *P*<.001), and those who preferred to receive written health information in English versus language other than English (30.38, SD 5.86 vs 22.22, SD 8.32; difference 8.17, 95% CI 5.45-10.8; *P*<.001; Table 1). There were also small but significantly higher scores in those free of hypertension or other chronic medical conditions.

### Mean eHEALS Scores by Web-Based Health Information Seeking, Interest in eHealth Strategies, and Use of Health and Fitness Apps

In total, 74.4% (224/301) of participants used the internet several times per day, 71.4% (215/301) of participants sought web-based health information, and 31.6% (94/297) of smartphone or tablet owners used health and fitness apps. Of the internet users, 84.4% (254/301) were interested in future eHealth interventions. As shown in Table 2, the total eHEALS scores were significantly higher in those who used the internet several times per day; those using the web for health information; those using social media, Twitter, YouTube, and health and fitness apps; and those who showed interest in future eHealth interventions.

**Table 2:** Mean overall eHEALS^a^ scores according to technology use.

		Values (n=301), n (%)	eHEALS score, mean (SD)	Difference in eHEALS score (95% CI)	*P* value^b^	
**Internet use frequency**						<.001^c^
	About once per day or less	74 (24.6)	26.49 (7.57)	Referent		
	Several times per day	224 (74.4)	30.24 (6.17)	3.76 (1.83 to 5.68)		
	Missing	3 (1)	N/A^d^	N/A		
**Internet use for health information**						.001^e^
	No	84 (27.9)	26.37 (7.48)	Referent		
	Yes	215 (71.4)	30.34 (6.23)	3.98 (2.16 to 5.78)		
	Missing	2 (0.7)	N/A	N/A		
**Social media use**						.007^e^
	No	68 (22.6)	26.97 (7.74)	Referent		
	Yes	231 (76.7)	30.00 (6.25)	3.03 (1.06 to 5.06)		
	Missing	2 (0.7)	N/A	N/A		
**Twitter use**						.03^e^
	No	248 (82.4)	28.93 (6.66)	Referent		
	Yes	50 (16.6)	31.48 (6.43)	2.55 (0.65 to 4.53)		
	Missing	3 (1)	N/A	N/A		
**YouTube**						.047^e^
	No	45 (15)	27.36 (7.56)	Referent		
	Yes	253 (84.1)	29.70 (6.49)	2.34 (0.04 to 4.81)		
	Missing	3 (1)	N/A	N/A		
**Interested in future eHealth support^f^**						.047^g^
	No	47 (15.6)	26.91 (9.15)	Referent		
	Yes	254 (84.4)	29.71 (6.25)	2.79 (0.3 to 5.57)		
**Health and fitness apps^h^**						.001^e^
	No	199 (67)	27.10 (8.07)	Referent		
	Yes	94 (31.6)	30.90 (6.90)	3.81 (2.07 to 5.73)		
	Missing	4 (1.3)	N/A	N/A		

^a^eHEALS: eHealth Literacy Scale.

^b^*P* value is for comparison of eHEALS scores.

^c^Welch *F test* on trimmed mean and Winsorized variance as data violated constant variance assumption and were far from normality.

^d^N/A: not applicable.

^e^The bootstrap method with 1000 repetitions was used, as the data differed substantially from the normal distribution. For the bootstrap method, bias-corrected and accelerated CIs were reported.

^f^The 6 different modes of eHealth support in the future, included (1) accessing a webpage including a forum where you could connect with others like you; (2) accessing a YouTube channel for people with your conditions that has experts talking about best management; (3) using a smartphone app or wearable device that can monitor your condition, track your progress on your health goals, or provide reminders about when to take your medications; (4) following a specific Twitter account for your conditions; (5) signing up for personalized text messages providing health updates or reminders for your conditions; or (6) using a web-based education program.

^g^Welch *t* test.

^h^App use by 297 smartphone or tablet owners.

### Characteristics Associated With eHealth Literacy: Quantile Regression

The results of the multivariable quantile regression using demographic, health status, and technology use variables for internet users are shown in Table 3. Language preference was the only variable independently associated with eHealth literacy. Expressing a preference for written health information in languages other than English reduced eHEALS scores by –6.0 (95% CI –9.61 to –2.39) points.

**Table 3:** Quantile regression of eHealth Literacy Scale scores through median for internet users (n=301).

			Internet users, coefficient (95% CI)
Age (years)			0.00 (–0.05 to 0.05)
**Gender**			
	Male	Referent	
	Female	0.50 (–0.47 to 1.47)	
**Marital status**			
	Not married	Referent	
	Married	–0.50 (–2.04 to 1.04)	
**Education**			
	<High school	Referent	
	High School	7.69 (–13.17 to 28.56)	
	≥College	10.69 (–10.17 to 31.55)	
**Lived in Canada (years)**			
	>5	Referent	
	0-5	–1.0 (–2.09 to 0.09)	
**South Asian community**			
	Sikh	Referent	
	Hindu	0.00 (–1.03 to 1.03)	
	Other	–0.69 (–2.34 to 0.96)	
**Confidence in filling out medical forms**			
	>Not at all	Referent	
	Not at all	–4.81 (–20.19 to 10.57)	
≥**1 chronic condition**			
	No	Referent	
	Yes	–0.50 (–1.49 to 0.49)	
**Language preference**			
	English	Referent	
	Not English	–6.0 (–9.61 to –2.39)	
**Diabetes**			
	No	Referent	
	Yes	–0.50 (–2.69 to 1.69)	
**Amount of internet use**			
	Once per day or less	Referent	
	Several times per day	0.50 (–1.34 to 2.34)	
**Internet use for health information**			
	No	Referent	
	Yes	1.00 (–0.58 to 2.58)	

## Discussion

### Principal Findings

Among our sample of primarily Sikh, highly educated internet users recruited from community pharmacies and family physician offices, the mean overall eHEALS score was 29.3 (SD 6.8) out of 40, suggesting high levels of eHealth literacy. Almost three quarters of the respondents agreed to at least 5 out of 8 eHEALS items and were categorized as having adequate eHealth literacy [49]. A large proportion of respondents (72%-78%) in this study reported knowing how to use the internet to answer health questions, how to use the health information found on the internet for support, and how to find helpful resources on the internet, whereas fewer (61%-67%) were confident in evaluating and using health information from the internet. Although there were statistically significant differences in eHEALS scores according to age, educational achievement, language preference, and the presence of hypertension or other chronic medical conditions, language preference was the only independent variable associated with eHealth literacy.

### Comparison With Prior Work

#### Level of eHealth Literacy

Most comparable with our study, is a case study conducted by Zibrik et al [59], which used quantitative and qualitative methods to explore eHealth literacy in a convenience sample of 896 established Chinese and Punjabi immigrant seniors recruited from public health events in British Columbia, Canada. Although overall and item-specific eHEALS scores were not reported in their paper, the authors concluded that their sample had low eHealth literacy levels compounded by challenges related to language, culture, attitude, and accessibility. The discrepancy between this conclusion and our results can be attributed to the fact that we focused our analysis on internet users and excluded eHEALS scores from internet nonusers, whereas Zibrik included all participants regardless of internet use status. Another Canadian survey assessing digital health literacy in South Asian women suggested high digital health literacy via high rates of mobile phone internet use but was only reported in abstract form [60].

Our results compare favorably to existing studies that looked at measures of eHealth literacy among internet users in the general population, reporting mean overall eHEALS scores ranging between 24 and 30 out of 40 [26,49,61-63]. For example, in a 2014 survey, Milne et al [49] reported an overall eHEALS score of 24.0 (SD not reported) among 83 primary lung cancer survivors at a cancer center in Toronto, Ontario, 78% of whom had access to e-resources. Only 34% of lung cancer survivors in Milne’s study agreed with 5 or more eHEALS items, whereas we found that 58% of all respondents agreed with 5 or more items [49]. More recently, James et al [63] reported a mean eHEALS score of 30.4 (SD 7.8) in a sample of 881 African American adults surveyed between April 2014 and January 2015 in North Central Florida.

In other studies focusing on eHealth literacy among internet users, as in our study, overall mean eHEALS scores ranged from 16.1 (SD 4.25) to 30.34 (SD 5.30), depending on the population studied [16,22,50,64-68]. Our finding that the items with the lowest level of agreement among internet users are related to their ability to evaluate the quality of web-based health resources is highly consistent with the findings of several other studies [63,65,66,69].

#### Variables Associated With eHealth Literacy

Several studies have explored variables associated with eHealth literacy [16,17,22,34,50,62,65]. The most commonly reported are age [16,22,50,65], education [16,65], and markers of frequency or degree of internet use [17,22,34,62]. However, other associations, including the number of devices used to access web-based health information [16] and language [65], have also been reported. Conflicting results led Richtering et al [17] to conclude that variables associated with eHealth literacy are largely dependent on population. For example, in their study examining eHealth literacy in 453 participants enrolled in a randomized controlled trial of consumer-focused eHealth for cardiovascular risk management in primary care in Australia, they showed that the frequency of internet use was the sole predictor of eHealth literacy [17]. Focusing specifically on underserved populations, a systematic review of the research suggests that internet use experience, urban dwelling, higher income, overall health literacy, and higher education are associated with higher eHealth literacy [34]. Notably, Zibrik et al reported that college-level education and female gender were associated with higher eHEALS scores in 545 Punjabi seniors included in their Canadian study [59].

By contrast, we found that language preference was the only variable independently associated with eHealth literacy. This could be a proxy for acculturation, as years living in Canada were not an independent predictor in our model. Limited English proficiency is a widely cited barrier in studies examining health care and eHealth access in immigrant or minority populations [59]. However, contradictory evidence exists in this area, where language was not found to be an independent predictor of eHealth literacy in a Canadian study by Milne [49]. However, being a non-English speaker was significantly associated with lower eHealth literacy in an American study that included a significant number of Hispanic and African American parents whose children have special health care needs [65]. Further, our work is in contrast to that of Neter et al, which suggests that language difficulties should manifest as lower health literacy, rather than eHealth literacy, as health literacy as a concept is anchored in a cultural and language context, whereas eHealth literacy is anchored in the empowering and capital-enhancing qualities of the internet [37]. Our finding that educational status was not associated with eHealth literacy may be a result of the highly educated survey sample.

### Implications for Practice and Research

First, although our results suggest that a large proportion of South Asian internet users have adequate eHealth literacy, there remains a sizable minority in our study group whose eHealth literacy can be strengthened. Understanding levels of eHealth literacy is even more important in 2022 than in 2014, as the COVID-19 pandemic has globally increased the remote internet-based delivery of health care [19]. Second, our results suggest that there is an opportunity to improve internet users’ ability to differentiate high- from low-quality health information. Beyond assessing web-based health information about diabetes, cardiovascular disease, physical activity, and diet or nutrition, the COVID-19 infodemic is another example that illustrates the need for individuals to be better able to identify medical misinformation on the internet [21]. Training individuals to recognize misinformation could be one way to increase their eHealth literacy. Another approach may include health care organizations and provider support for patients to navigate and access high-quality and reliable web-based health information and resources via social media [70]. Finally, given that language preference was the only predictor of eHealth literacy, and this is likely a nonmodifiable factor, we suggest that interventions to improve eHealth literacy should be targeted toward those who have low English language proficiency and delivered in the individual’s preferred language. Strategies involving peers, friends, and family members may also be effective.

Our study suggests several areas for future research. First, further work with representative samples should compare levels of health and eHealth literacy using newly available tools among recent and established Canadian immigrants and nonimmigrants similar to that of Neter et al [37]. Second, work should be done to develop theory driven, cultural, and language-tailored interventions to increase the uptake of eHealth interventions and improve eHealth literacy. Finally, high-quality randomized controlled trials are necessary to evaluate theory-based eHealth literacy interventions in ethnocultural minority and immigrant populations, and their impact on health outcomes.

### Strengths and Limitations

At the time the survey was conducted in 2014, our work was unique, and to our knowledge, there are only 2 other published studies exploring eHealth literacy in members of the South Asian community in Canada [59,60]. Despite this, our study has several limitations. First, our data were collected in 2014 and are not likely to reflect the current use of digital health technologies or eHealth literacy. Second, as nonprobability sampling was used and only a subset of the entire survey sample completed the eHEALS assessment, we were unable to generalize our results to the larger South Asian population, as the sample is not representative. Furthermore, our results primarily pertain to the English- and Punjabi-speaking Sikh community, as we did not translate our survey into other commonly spoken languages (eg, Hindi and Urdu). Third, we did not formally validate the Punjabi version of the eHEALS, and there may be issues with conceptual translation and some variability in administration. However, few eHealth literacy assessments have been completed in Punjabi, and we used established procedures for translating survey items. We reported high internal consistency for the 8 items in this survey, and we had 2 trained research assistants who administered over 65% of the surveys. Fourth, eHEALS measures perceived eHealth literacy rather than actual eHealth literacy or skills as measured by performance tests [67,71]. In addition, it is worth noting that the tool does not address the ability to use Web 2.0 functionalities such as social media, mobile devices, and health and fitness apps for health behavior change purposes as do new tools [72]. Fifth, as our survey was primarily administered in person, social desirability bias may overinflate reported eHEALS scores and estimates of device ownership, internet use, and willingness to use future eHealth tools, whereas self-reporting may introduce recall bias in outcome and demographic variables. Finally, we recognize that the question relating to language preference for written health information could have been improved by asking about the primary language spoken in the home and that the use of a single health literacy screening question rather than a full health literacy questionnaire is not optimal.

### Conclusions

This cross-sectional study in a subset of e-Patient Project survey respondents provides insights for clinicians and researchers on the levels and variables associated with eHealth literacy in a sample of South Asian adults living in a major Canadian city. Preferring written health information in languages other than English was the only independent variable associated with reduced eHealth literacy in our sample. Our results suggest that respondents may still benefit from interventions targeting skills to evaluate web-based health resources and that linguistically and culturally appropriate interventions are required to improve eHealth literacy.

## Appendix

Multimedia Appendix 1 English version of the e-Patient Project Survey and eHealth Literacy Scale.

Multimedia Appendix 2 Punjabi translation of the eHealth Literacy Scale.

Multimedia Appendix 3 eHealth Literacy Scale mean item scores, scale reliability, and principal component analysis (n=301).

Multimedia Appendix 4 Characteristics of internet nonuser eHealth Literacy Scale completers (n=83).

## Abbreviations

eHEALS: eHealth Literacy Scale
mHealth: mobile health
