# Supplementary material for: eHealth Literacy in a Sample of South Asian Adults in Edmonton, Alberta, Canada: Subanalysis of a 2014 Community-Based Survey
Source: JMIR Form Res. 2022 Mar 30;6(3):e29955. doi: 10.2196/29955 (PMC9008520; doi:10.2196/29955)
Supplement: Multimedia Appendix 2 [file formative_v6i3e29955_app2.pdf]

ਪ੍ਰਸ਼ਨ 23 : ਸਿਹਤ ਦੀ ਜਾਣਕਾਰੀ ਲਈ ਇੰਟਰਨੈੱਟ ਕਿੰਨਾ ਜਰੂਰੀ ਲੱਗਦਾ ਹੈ?

0 ਬਿਲਕੁਲ ਜਰੂਰੀ ਨਹੀਂ (1)

0 ਜਰੂਰੀ ਨਹੀਂ (2)

0 ਪੱਕਾ ਪਤਾ ਨਹੀਂ (3)

0 ਜਰੂਰੀ ਹੈ (4)

0 ਬਿਲਕੁਲ ਜਰੂਰੀ ਹੈ (5)

0 ਕੋਈ ਉਤਰ ਨਹੀਂ (ਇਸ ਨੂੰ ਨਾ ਪੜ੍ਹੋ) (6)

ਪ੍ਰਸ਼ਨ 24: ਇੰਟਰਨੈੱਟ ਸਿਹਤ ਸਿਹਤ ਸਬੰਧੀ ਫੈਸਲੇ ਕਰਨ ਵਿੱਚ ਕਿੰਨੀ ਮਦਦ ਕਰਦਾ ਹੈ?

0 ਬਿਲਕੁਲ ਲਾਭਦਾਇਕ ਨਹੀਂ (1)

0 ਲਾਭਦਾਇਕ ਨਹੀਂ (2)

0 ਪੱਕਾ ਪਤਾ ਨਹੀਂ (3)

0 ਲਾਭਦਾਇਕ ਹੈ (4)

0 ਬਹੁਤ ਲਾਭਦਾਇਕ ਹੈ (5)

0 ਕੋਈ ਉਤਰ ਨਹੀਂ (ਇਸ ਨੂੰ ਨਾ ਪੜ੍ਹੋ) (6)

ਪ੍ਰਸ਼ਨ ੨੫: ਤੁਸੀਂ ਹੇਠ ਲਿਖੇ ਬਿਆਨਾਂ ਨਾਲ ਕਿਸ ਹੱਦ ਤਕ ਸਹਿਮਤ ਜਾਂ ਅਸਹਿਮਤ ਹੋ?

ਕੰਮ	ਬਿਲਕੁਲ ਸਹਿਮਤ ਨਹੀਂ (1)	ਸਹਿਮਤ ਨਹੀਂ (2)	ਪੱਕਾ ਪਤਾ ਨਹੀਂ (3)	ਸਹਿਮਤ ਹਾਂ (4)	ਬਿਲਕੁਲ ਸਹਿਮਤ ਹਾਂ (5)	ਕੋਈ ਉਤਰ ਨਹੀਂ। (ਇਸ ਨੂੰ ਨਾ ਪੜ੍ਹੋ) (6)
ਮੈਨੂੰ ਪਤਾ ਹੈ ਕਿ ਸਿਹਤ ਸਬੰਧੀ ਇੰਟਰਨੈਟ 'ਤੇ ਕਿਹੜੇ ਸਾਧਨ ਉਪਲੱਬਧ ਹਨ। (੧)	µ	µ	µ	µ	µ	µ
ਮੈਨੂੰ ਪਤਾ ਹੈ ਕਿ ਸਿਹਤ ਸਬੰਧੀ ਮਦਦਗਾਰ ਸਾਧਨ ਇੰਟਰਨੈਟ 'ਤੇ ਕਿਥੋਂ ਲੱਭਣੇ ਹਨ। (੨)	µ	µ	µ	µ	µ	µ
ਮੈਨੂੰ ਪਤਾ ਹੈ ਕਿ ਸਿਹਤ ਸਬੰਧੀ ਮਦਦਗਾਰ ਸਾਧਨ ਇੰਟਰਨੈਟ 'ਤੇ ਕਿਵੇਂ ਲੱਭਣੇ ਹਨ। (੩)	µ	µ	µ	µ	µ	µ
ਮੈਨੂੰ ਪਤਾ ਹੈ ਕਿ ਆਪਣੀ ਸਿਹਤ ਬਾਰੇ ਸਵਾਲਾਂ ਦੇ ਜਵਾਬ ਲੱਭਣ ਲਈ ਇੰਟਰਨੈਟ 'ਤੇ ਕਿਵੇਂ ਜਾਣਾ ਹੈ। (੪)	µ	µ	µ	µ	µ	µ
ਮੈਨੂੰ ਪਤਾ ਹੈ ਕਿ ਸਿਹਤ ਸਬੰਧੀ ਜਿਹੜੀ ਜਾਣਕਾਰੀ ਇੰਟਰਨੈਟ ਤੋਂ ਪ੍ਰਾਪਤ ਕੀਤੀ ਹੈ, ਉਸਨੂੰ ਆਪਣੇ ਲਈ ਕਿਵੇਂ ਵਰਤਣਾ ਹੈ। (੫)	µ	µ	µ	µ	µ	µ
ਮੈਂ ਇੰਟਰਨੈਟ ਵਾਲੇ ਸਿਹਤ ਸਬੰਧੀ ਸਾਧਨਾਂ ਦੀ ਪਰਖ ਕਰ ਸਕਦਾ ਹਾਂ। (੬)	µ	µ	µ	µ	µ	µ
ਮੈਂ ਦੱਸ ਸਕਦਾ ਜਾਂ ਕਿ ਇੰਟਰਨੈਟ 'ਤੇ ਕਿਹੜੇ ਸਾਧਨ ਭਰੋਸੇਯੋਗ ਹਨ ਅਤੇ ਕਿਹੜੇ ਨਹੀਂ। (੭)	µ	µ	µ	µ	µ	µ
ਮੈਨੂੰ ਸਿਹਤ ਸਬੰਧੀ ਇੰਟਰਨੈਟ ਤੋਂ ਲਈ ਜਾਣਕਾਰੀ ਨੂੰ ਵਰਤਣ ਵਿੱਚ ਭਰੋਸਾ ਹੈ। (੮)	µ	µ	µ	µ	µ	µ
