# Supplementary material for: eHealth Literacy in a Sample of South Asian Adults in Edmonton, Alberta, Canada: Subanalysis of a 2014 Community-Based Survey
Source: JMIR Form Res. 2022 Mar 30;6(3):e29955. doi: 10.2196/29955 (PMC9008520; doi:10.2196/29955)
Supplement: Multimedia Appendix 3 [file formative_v6i3e29955_app3.docx]

**Multimedia Appendix** 3**. eHealth Literacy Scale mean item scores, scale reliability, and principal component analysis (n=301)**

|  | **Mean** | **SD** | **Factor loading** | **Corrected item – Total correlation** |
| --- | --- | --- | --- | --- |
| 1. I know what health resources are available on the internet | 3.56 | 1.02 | 0.73 | 0.80 |
| 2. I know where to find helpful health resources on the internet | 3.67 | 0.97 | 0.77 | 0.84 |
| 3. I know how to find helpful health resources on the internet | 3.72 | 0.98 | 0.78 | 0.84 |
| 4. I know how to use the internet to answer my questions about health | 3.83 | 0.99 | 0.72 | 0.80 |
| 5. I know how to use the health information I find on the internet to help me | 3.77 | 0.93 | 0.80 | 0.85 |
| 6. I have the skills I need to evaluate the health resources I find on the internet | 3.64 | 1.00 | 0.78 | 0.85 |
| 7. I can tell high quality health resources from low quality health resources on the internet | 3.50 | 1.06 | 0.69 | 0.78 |
| 8. I feel confident in using information from the internet to make health decisions. | 3.57 | 1.00 | 0.68 | 0.78 |
|  |  |  |  |  |
| eHEALS score | 29.27 | 6.84 |  |  |
| Eigenvalue first component | 5.95 |  |  |  |
| Variance accounted for | 74.4% |  |  |  |
| Cronbach alpha | 0.950 |  |  |  |
