# Supplementary material for: eHealth Literacy in a Sample of South Asian Adults in Edmonton, Alberta, Canada: Subanalysis of a 2014 Community-Based Survey
Source: JMIR Form Res. 2022 Mar 30;6(3):e29955. doi: 10.2196/29955 (PMC9008520; doi:10.2196/29955)
Supplement: Multimedia Appendix 4 [file formative_v6i3e29955_app4.docx]

**Multimedia Appendix 4. Characteristics of Internet nonuser eHealth Literacy Scale completers (n=83)**

**Table 1. Demographics**

| **Demographic** | **Values (n=83)**  **n (%)** |
| --- | --- |
| **Age Mean (SD)** | 61.85 (14.87) |
| **Age (years)** |  |
| <65 | 38 (45.8) |
| ≥65 | 44 (53.0) |
| Missing | 1 (1.2) |
| **Sex** |  |
| Male | 40 (48.2) |
| Female | 43 (51.8) |
| **Marital status** |  |
| Not married | 9 (12.5) |
| Married | 69 (87.5) |
| Missing | 5 (6.0) |
| **Education** |  |
| <High school | 21 (25.3) |
| High school | 34 (41.0) |
| ≥College | 24 (28.9) |
| Missing | 4 (4.8) |
| **Lived in Canada (years)** |  |
| >5 | 67 (80.7) |
| 0-5 | 14 (16.9) |
| Missing | 1 (1.2) |
| **South Asian community** |  |
| Sikh | 71 (85.5) |
| Hindu | 6 (7.2) |
| Other | 5 (6.0) |
| Missing | 1 (1.2) |
| **Confidence in filling out medical forms** |  |
| >Not at all | 62 (74.7) |
| Not at all | 21 (25.3) |
| **Health status** |  |
| Excellent | 12 (14.5) |
| Very Good | 19 (22.9) |
| Good | 31 (37.3) |
| Fair | 12 (14.5) |
| Poor | 8 (10.8) |
| **Diabetes** |  |
| No | 58 (67.5) |
| Yes | 25 (30.1) |
| Missing | 2 (2.4) |
| **High blood pressure** |  |
| No | 39 (47.0) |
| Yes | 43 (51.8) |
| Missing | 1 (1.2) |
| **Heart disease** |  |
| No | 70 (84.3) |
| Yes | 11 (13.3) |
| Missing | 2 (2.4) |
| **Lung conditions** |  |
| No | 73 (88.0) |
| Yes | 7 (8.4) |
| Missing | 3 (3.6) |
| **Arthritis** |  |
| No | 54 (65.1) |
| Yes | 27 (32.5) |
| Missing | 1 (1.2) |
| **Cancer** |  |
| No | 77 (92.8) |
| Yes | 3 (3.6) |
| Missing | 3 (3.6) |
| **Other chronic condition** |  |
| No | 67 (80.7) |
| Yes | 14 (16.9) |
| Missing | 2 (2.4) |
| **High Cholesterol** |  |
| No | 44 (53.0) |
| Yes | 26 (31.3) |
| Missing | 13 (15.7) |
| **≥1 Chronic condition** |  |
| No | 19 (22.9) |
| Yes | 64 (77.1) |
| **Language preference** |  |
| English | 24 (28.9) |
| Not English | 56 (67.5) |
| Missing | 3 (3.6) |
| **Location of Recruitment** |  |
| Community setting | 13 (15.7) |
| Health setting | 56 (67.5) |
| Missing | 3 (3.6) |

**Table 2. Supplemental eHEALS Item Responses (n=83)**

|  | **Mean (SD)** | **Important** | **Undecided** | **Not Important** |
| --- | --- | --- | --- | --- |
| How **important** is it for you to be able to access health resources on the internet | 2.88 (1.22) | 30 (36.1) | 19 (22.9) | 34 (41.0) |
|  | **Mean (SD)** | **Useful** | **Undecided** | **Not Useful** |
| How **useful** do you feel the internet is in helping you in making decisions about your health? | 2.57 (1.24) | 23 (27.7) | 22 (26.5) | 38 (45.8) |

**Table 3. Individual eHEALS Item Responses. Data for strongly disagree and disagree was collapsed as was agree and strongly agree (n=83)**

|  | **Mean (SD)** | **Agree** | **Undecided** | **Disagree** |
| --- | --- | --- | --- | --- |
| I know what health resources are available on the internet | 1.95 (1.06) | 9 (10.8) | 14 (16.9) | 60 (72.3) |
| I know where to find helpful health resources on the Internet | 1.88 (0.97) | 5 (6.0) | 16 (19.3) | 62 (74.7) |
| I know how to find helpful health resources on the internet | 1.90 (0.97) | 5 (6.0) | 17 (20.5) | 61 (73.56) |
| I know how to use the internet to answer my questions about health | 2.0 (1.09) | 9 (10.8) | 16 (19.3) | 58 (69.9) |
| I know how to use the health information I find on the internet to help me | 2.20 (1.09) | 12 (14.5) | 20 (24.1) | 51 (61.4) |
| I have the skills I need to evaluate the health resources I find on the internet | 2.11 (1.2) | 14 (16.9) | 16 (19.3) | 53 (63.9) |
| I can tell high-quality health resources from low-quality health resources on the internet | 2.07 (1.19) | 12 (14.5) | 16 (19.3) | 55 (66.3) |
| I feel confident in using information from the internet to make health decisions | 2.18 (1.12) | 12 (14.5) | 18 (21.7) | 53 (63.9) |
| **Overall score** | 16.30 (7.81) |  |  |  |
